# Endoscopic sleeve gastroplasty using the novel single‐channel suturing device: A multicenter experience

**DOI:** 10.1002/deo2.213

**Published:** 2023-02-23

**Authors:** Ravishankar Asokkumar, Rajesh Ravi, Voraboot Taweerutchana, Yu Bin Tan, Kotchakorn Maipang, Lim Chin Hong, Nicha Srisuworanan, Lee Phong Ching, Christopher Khor, Jason Chang, Nonthalee Pausawasdi

**Affiliations:** ^1^ Department of Gastroenterology and Hepatology Singapore General Hospital Singapore; ^2^ DUKE‐NUS Graduate Medical School Singapore; ^3^ Department of Surgery, Division of General Surgery, Minimally Invasive Surgery Unit, Faculty of Medicine Siriraj Hospital Mahidol University Bangkok Thailand; ^4^ Siriraj GI Endoscopy Center, Faculty of Medicine Siriraj Hospital Mahidol University Bangkok Thailand; ^5^ Department of Upper Gastrointestinal and Bariatric Surgery, Division of Surgery Singapore General Hospital Singapore; ^6^ Department of Endocrinology Singapore General Hospital Singapore; ^7^ Department of Medicine, Division of Gastroenterology, Faculty of Medicine Siriraj Hospital Siriraj GI Endoscopy Center, Mahidol University Bangkok Thailand

**Keywords:** bariatric endoscopy, endoscopic gastroplasty, ESG, obesity, weight loss

## Abstract

**Background and aim:**

Endoscopic sleeve gastroplasty (ESG) is an effective treatment for obesity. Recently, a novel single‐channel endoscopic suturing device has been made available to overcome the need for a double‐channel endoscope. However, there is limited evidence evaluating its utility for ESG. In this multicenter study, we aim to assess the efficacy and safety of the single‐channel suturing device for ESG.

**Methods:**

We reviewed the records of 18 patients who underwent ESG using the novel device at the Singapore General Hospital, Singapore, and Siriraj Hospital, Bangkok, between 2020–2021. We adopted a “U” suture pattern. Our primary outcome was to assess technical feasibility and safety. The secondary outcome was to determine the percentage of total body weight loss at 1 year.

**Results:**

The mean ± SD age and body mass index were 42 ± 8.5 years and 34.9 ± 4.4 kg/m^2^, respectively. The majority were female (61%). ESG was technically successful in 94% (*n* = 17) of patients. Device dislodgement occurred in one patient. We used an average of five sutures (range, 4–8), and the mean ± SD procedure time was 96.5 ± 43.8 min. No complications occurred. The mean ± SD length of stay was 2.3 ± 1.5 days. The mean ± SD percentage of total body weight loss at 6 and 12 months were 16 ± 5.2% and 13.1 ± 5.8%, respectively. We found that >5%, >10%, and >15% total body weight loss was observed in 83.3%, 72.2%, and 56%, respectively.

**Conclusion:**

ESG using the single‐channel endoscopic suturing system is safe and effective for inducing weight loss at 1 year in patients with obesity.

## INTRODUCTION

Obesity is a chronic progressive disease with a significant impact on patient's health and the cost of healthcare.^1^, ^2^, ^3^ Newer effective options are needed to combat the rising pandemic.^4^, ^5^ Endoscopic bariatric therapies have emerged as an effective approach to treating obesity. Unlike invasive bariatric surgeries, the ease of delivery and the reversible nature of endoscopic bariatric therapies would make them a patient‐preferred and widely adoptable treatment option for obesity.^6^ Among the endoscopic bariatric therapies, endoscopic sleeve gastroplasty (ESG) using the Overstitch device (Apollo Endosurgery, USA) has gained prominence because of its excellent safety and efficacy data. In a meta‐analysis that included 1815 patients, the average pooled total body weight loss (TBWL) at 12 months was 17.1 (95% CI: 15.1–19.1).^7^ Likewise, a study comparing ESG to laparoscopic sleeve and laparoscopic greater curve plication showed ESG induced an adjusted mean %TBWL of 18.5% at 2 years.^8^ Although the results are very promising, some of the challenges faced in scaling ESG is the lack of availability of double‐channel endoscopes in most endoscopic units for performing the procedure.

Recently, a novel suturing device (Overstitch Sx, Apollo Endosurgery) with a unique design has been made available to overcome this barrier. The device can be mounted on the most commercially available single‐channel endoscopes to perform endoscopic suturing and tissue opposition.^9^ However, there is only scarce data demonstrating its efficacy and safety for performing complex suturing involved with ESG. We hypothesize that the novel single‐channel device's technical aspects and suturing ability should be similar to the earlier generation double‐channel suturing device in performing ESG. In this multicenter study, we aim to evaluate the technical success, efficacy, and safety of the novel single‐channel suturing device for ESG.

## METHODS

### Trial design

We retrospectively reviewed the records of patients who underwent ESG using the single‐channel endoscopic suturing device at the Obesity Centers at Singapore General Hospital (*n* = 11), Singapore, and Siriraj Hospital (*n* = 7), Bangkok, Thailand, between November 2020 and August 2021. The institutional review board approved the study. All the patients consented to the procedure.

### Participants

Eighteen patients underwent ESG using the single‐channel device. All these patients had declined surgery and failed diet and lifestyle therapy. The inclusion criteria for ESG were (i) age ≥18 years, (ii) body mass index (BMI) ≥27.5 kg/m^2^, and (iii) being able to comply with instructions and provide informed consent.^10^ We excluded those with: (i) severe systemic illnesses, (ii) substance abuse, (iii) uncontrolled eating disorder, (iv) pregnancy, and (v) coagulopathy. We offered ESG to all and did not preferentially select patients for this novel device. We collected information on technical outcomes, length of stay, complication rates, and weight loss outcomes. We acknowledge that some of the patients in the Singapore group were used in our previous research describing our experience with ESG.^11^

## INTERVENTION

### Single‐channel suturing device

**FIGURE 1:**
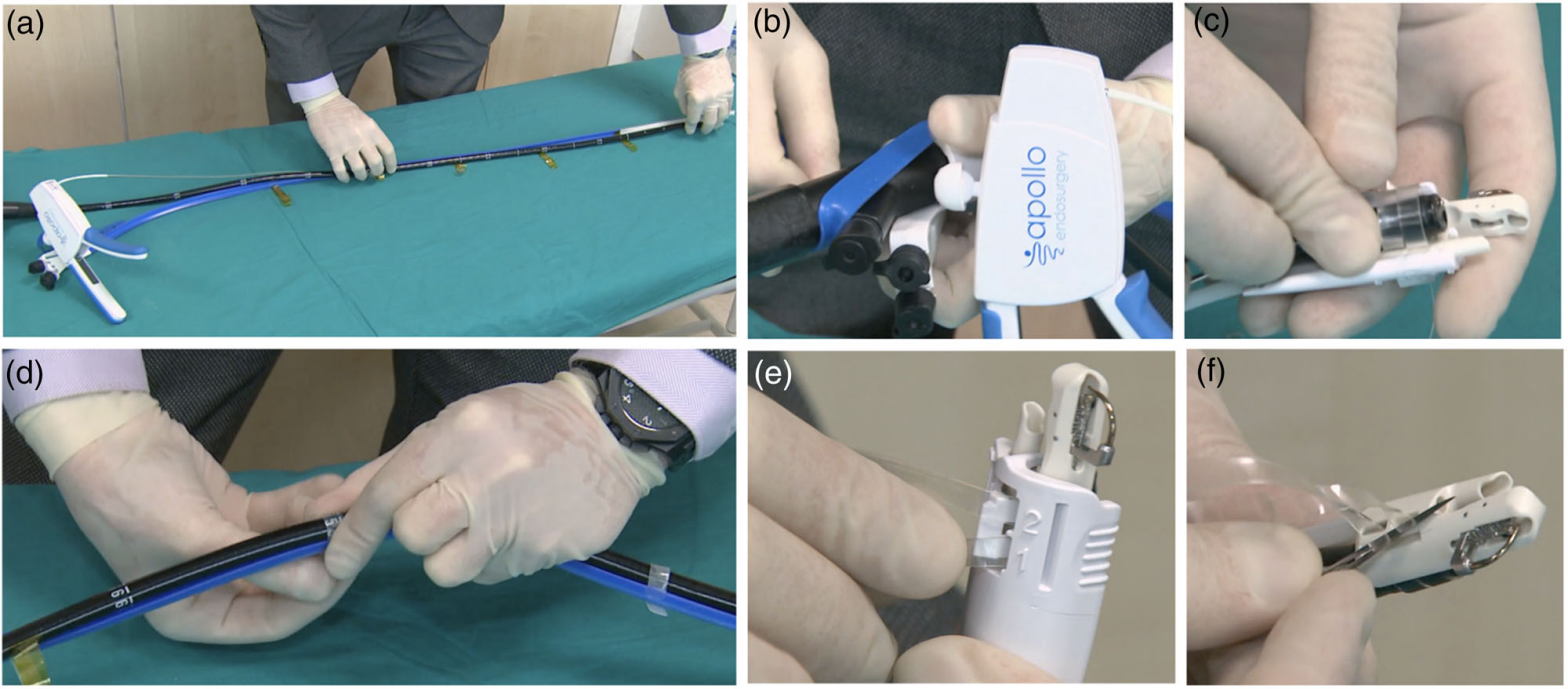
Assembly of the single‐channel suturing device. (a) Introduce the scope between the metallic wire and the plastic sheath and align across the length of the external catheter. (b) The plastic sheath has two external working channels. Fix them close to the endoscope channel, and secure them with the rubber strap. (c) Introduce the distal end of the scope into the plastic endcap. (d) Secure the plastic sheath to the scope shaft using silicone straps. (e) At the distal end, secure the endcap to the scope by tightening the straps. Make certain the tip of the endoscope is below the stop indicator in the endcap and the scope channel is near it. Tighten the straps in sequence and avoid overlap or gaps. Once done, remove the plastic case. (f) Trim the excess strap using a blade and smooth the cut edges.

The overstitch Sx device is compatible with single‐channel endoscopes with diameters of 8.8–9.8 mm from different endoscopy platforms. The device has an endcap with the needle driver and two external independent working channels for accessories. The endcap and the external working channels are fastened to the endoscope shaft using silicone straps. The suturing handle is secured to the scope channel using rubber straps (Figure 1). We have previously described the technique of assembling the single‐channel suturing device into the endoscope.^12^

### Single‐channel ESG

**FIGURE 2:**
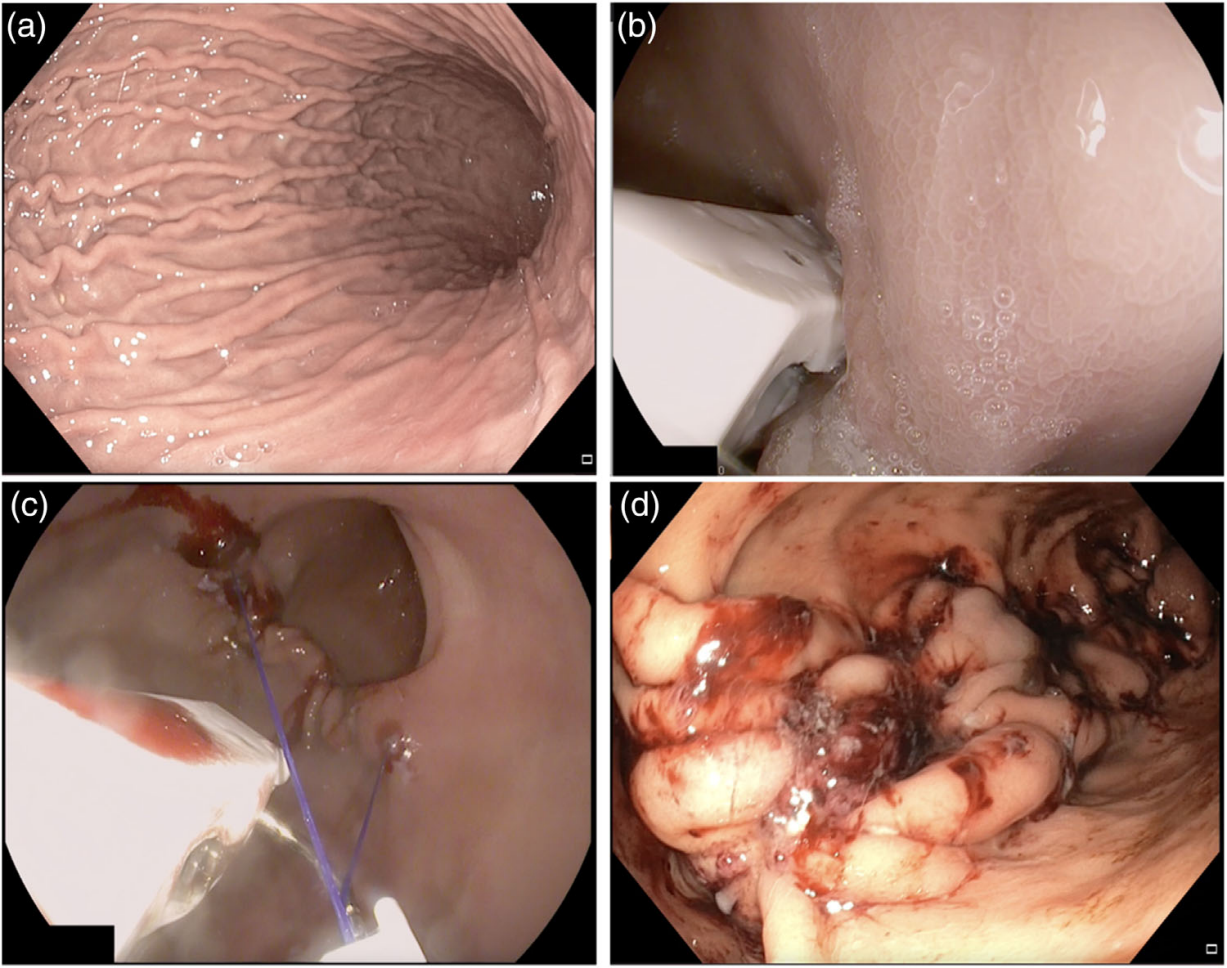
Endoscopic sleeve gastroplasty (ESG) using the single‐channel device. (a) Normal stomach before ESG. (b) Tissue is captured and pulled into the endcap to commence suturing. (c) Continuous suturing was performed following a “U” shaped suture pattern. (d) Tubular stomach appearance after ESG. The fundus is spared.

All ESG procedures (Overstitch Sx; Apollo Endosurgery) were done by three endoscopists (Ravishankar Asokkumar, Voraboot Taweerutchana, and Nonthalee Pausawasdi) with experience in endoscopic suturing and performing ESG. We performed the procedure using CO_2_ insufflation and under general anesthesia. We introduced the endoscope mounted with the single‐channel suturing device without using an overtube. Routine marking of the stomach with argon plasma coagulation was not performed as we found it ineffective in our practice. We started suturing at the distal body, slightly above the incisura and adopted a U‐shaped suture pattern comprising 8 to 10 full‐thickness bites per row. We performed 4–6 rows of suturing to collapse the greater curvature of the gastric body within the sutures. We did not routinely place reinforcing sutures (Figure 2) and spared the fundus.^11^, ^12^ Upon completion, we inspected the sleeve integrity and secured hemostasis. We monitored the patients for 24 hours and discharged them with antiemetics and proton pump inhibitors.

### Post‐procedure follow‐up

The follow‐up instructions and diet/exercise recommendations were similar between the two centers. The patients were followed up at regular intervals (biweekly in the initial phase and then monthly) by a multidisciplinary team comprising dieticians, gastroenterologists, and exercise therapists. The energy requirement was calculated from the Harris‐Benedict formula, considering the patients’ physical activity status. An energy deficit of about 2.6 MJ/day was prescribed to induce an approximate loss of between 0.5 and 1 kg/week. In the first month, we maintained the patients on a strict liquid diet (4 weeks), which included commercial meal replacement options or homemade soups. We progressively advanced to semi‐solid and solid food based on patient tolerance. We encouraged protein intake (1.5 g/kg/day) to prevent lean muscle mass.^11^, ^13^ In the first two weeks post‐ESG, we encouraged patients to walk and restrict techniques that increased intra‐abdominal pressure. Subsequently, depending on the patient's capacity, we devised an individualized exercise plan, such as a treadmill, stair‐climbing, jumping jacks, and squats (approximately 30–45 min/day).

We performed elective endoscopy at 1 month in seven patients to assess the sleeve integrity.

### Outcomes

The primary objective was to assess the technical feasibility and safety of the single‐channel suturing device for obesity. The secondary outcome was to determine the %TBWL at 12 months after ESG. We graded the adverse events using the Clavien‐Dindo classification.^14^

### Statistical methods

We expressed the continuous variables as mean ± SD or median (range) and categorical variables as percentages. We assessed for normality using the Shapiro‐Wilk test. We used the repeated measures analysis of variance test to compare weight loss outcomes at baseline and the last follow‐up after ESG. Post‐hoc paired t‐test test with Bonferroni correction was performed to evaluate the difference between the pairs. A *p*‐value of < 0.05 was considered to be significant.

## RESULTS

### Patient characteristics

Eighteen patients underwent ESG during the study period. Table 1 details the patients' characteristics. The mean ± SD age was 42 ±  8.5 years. The mean ± SD initial body weight and BMI were 93.3 ± 17.0 kg and 34.9 ± 4.4 kg/m^2^, respectively. The majority were female (61%, *n* = 11). One or more obesity‐related comorbidities were present in all our patients. Three (16%) patients were lost to follow at 1 year.

**TABLE 1:** Clinical characteristics of the study participants.

	**Patients (*n* = 18)**
Age ± SD, years (range)	42.0 ± 8.5
Female, *n* (%)	11 (61%)
Mean ± SD initial weight, kg	93.3 ± 17.0
Mean ± SD initial BMI, kg/m^2^	34.9 ± 4.4
Class I (≥27.5–32.4 kg/m^2^)	8 (44%)
Class II (32.5–37.4 kg/m^2^)	5 (28%)
Class III (≥37.5 kg/m^2^)	5 (28%)
Ethnicity, *n* (%)	
Chinese	6 (33%)
Indian	3 (17%)
Malay	2 (11%)
Thai	7 (39%)
Comorbid illness, *n* (%)	
Hypertension	7 (39%)
Fatty liver	5 (28%)
Hyperlipidemia	7 (39%)
Obstructive sleep apnea	7 (39%)
Diabetes mellitus	4 (22%)
Coronary artery disease	1 (0.6%)
Completed 6‐months, *n* (%)	15 (83%)
Completed 12‐months, *n* (%)	10 (56%)
Completed 1‐month, *n* (%)	18 (100%)
Follow‐up loss, *n* (%)	3 (17%)

### Technical outcomes and safety

The procedure was technically successful in 17 patients (94%; Table 2). In one patient, the silicone straps holding the endcap were severed, and the endcap was dislodged from the scope tip halfway through the procedure. The damaged device was removed, and a new single‐channel suturing system was used to complete the procedure, making it technically successful in all patients (100%). We used an average of five sutures (range 4–8) and adhered to the U‐shaped pattern in all patients. The mean procedural time was 96.5 ± 43.8 min. We did not encounter any suture breakage during the procedure. The mean ± SD length of stay was 2.3 ± 1.5 days. Immediate post‐procedure pain occurred in 8 patients (44%). The patients were monitored longer and responded to symptomatic treatment. All were asymptomatic at the time of discharge. The average decline in hemoglobin after the procedure was 0.5 ± 0.8 g/dl. No complications occurred after ESG.

**TABLE 2:** Outcomes of endoscopic sleeve gastroplasty using the single‐channel suturing device.

	**Patients (*n* = 18)**
Technical success, *n* (%)	17 (94%)
Mean sutures (range)	5 (4–8)
Adherence to the “U” pattern, *n* (%)	18 (100%)
Mean ± SD procedure time, min	96.5 ± 43.8
Mean ± SD length of stay, days	2.3 ± 1.5
Post‐procedure pain, *n* (%)	8 (44%)
Vomiting, *n* (%)	0 (0%)
Major complications	Nil
Mean ± SD hemoglobin change (g/dl)	0.5 ± 0.8

### Weight loss outcome

Among the study cohort, 15 (83%) completed 6 months, and 10 (56%)  completed 12 months. Rest are at different phases of follow‐up. Three patients were lost to follow‐up. Two at 1 month and the other at 5 months. The overall mean ± SD TBWL, %TBWL, and BMI loss at 6 months were 14.8 ± 5.5 kg, 16 ± 5.2%, and −5.4 ± 1.7 kg/m^2^, respectively. At 12 months, the mean ± SD TBWL, %TBWL, and BMI loss were 12 ± 5.8 kg, 13.1 ± 5.8%, and −4.4 ± 1.9 kg/m^2^, respectively. Pairwise comparison showed no significant difference in weight loss between 6 and 12 months (Table 3). We found that > 5%, > 10%, and > 15% TBWL was observed in 83.3%, 72.2%, and 56%, respectively. Among the patients lost to follow‐up, the mean ± SD %TBWL at the last visit was 6.4 ± 3.2%.

**TABLE 3:** Weight loss outcome after endoscopic sleeve gastroplasty.

**Outcomes**	**1‐month**	**6‐month**	**12‐month**	***p*‐Value**
Mean ± SD TBWL, kg	8.2 ± 3.1	14.8 ± 5.5	12 ± 5.8	0.008
Mean ± SD %TBWL	8.9 ± 3	16 ± 5.2	13.1 ± 5.8	0.004
Mean ± SD ΔBMI, kg/m^2^	−3.1 ± 1.1	−5.4 ± 1.7	−4.4 ± 1.9	0.005

Pairwise comparison at 6 and 12 months showed no difference in outcomes. Abbreviations: ΔBMI, change in body mass index; %TBWL, percentage of total body weight loss.

We noticed an improvement in obesity‐related comorbidities in the study population. Among the patients with hypertension (*n* = 7), 43% showed more than 10 mm Hg reduction in systolic blood pressure. In patients with fatty liver, a reduction in the aspartate aminotransferase levels was observed during follow‐up (83 ± 68 IU/L vs. 42 ± 34 IU/L). In patients with diabetes mellitus, we observed a reduction in the HbA1C (7.06 ± 0.5% vs. 5.4 ± 0.7%).

Endoscopy performed at 1 month in seven patients (Thailand) showed intact sleeve contour and no suture disruption. The lumen restriction effect was well maintained.

## DISCUSSION

This is the first multicenter study demonstrating the utility of a single‐channel suturing system for bariatric applications. We showed that ESG performed using the single‐channel suturing device is technically feasible and safe. All patients recovered without significant complications and only required a short hospital stay. The procedure induced considerable weight loss at 12 months and improved comorbid illnesses.

ESG involves suturing the stomach and imbricating the greater curvature to reduce gastric volume significantly.^15^ The restricted gastric volume limits food intake, reduces gastric emptying, induces satiation and early satiety, and results in significant weight loss.^16^, ^17^ A multicenter randomized study showed that ESG induced a TBWL of 13.6% compared to 0.8% in the lifestyle group at 52 weeks.^18^ In addition, 68% maintained the weight loss at 2 years, and 80% experienced an improvement in one or more metabolic comorbidities after ESG. The serious adverse event rate was 2%. The randomized study results and the available evidence on ESG have firmly positioned it as an effective option for obesity.^18^, ^19^ The excellent safety profile and the minimally invasive nature raise the possibility of ESG becoming the first‐line approach before considering bariatric surgery in a select group of patients. Besides, Al Qahtani et al. have demonstrated that bariatric surgery can be safely performed after ESG in patients who failed to achieve significant weight loss.^20^ The second‐generation suturing device is the predominantly used system for performing ESG. While efforts are being made to train more endoscopists and scale ESG as a weight loss option, one of the several barriers is the lack of widespread availability of double‐channel endoscopes in most units. The single‐channel endoscopic suturing system attempts to overcome this obstacle by making it compatible with most upper endoscopes, thus negating the need for additional investments to procure a double‐channel endoscope.^9^

**FIGURE 3:**
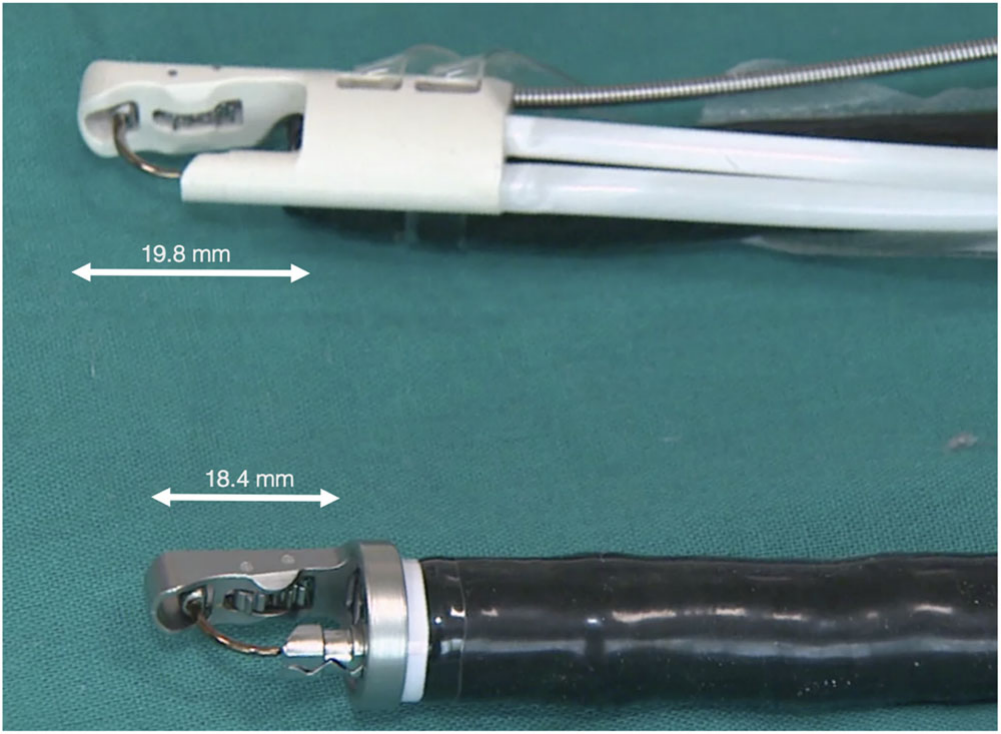
Comparison of the endcap design of the single and double‐channel suturing system. The tower height is longer in a single‐channel suturing device compared to the double‐channel device.

**FIGURE 4:**
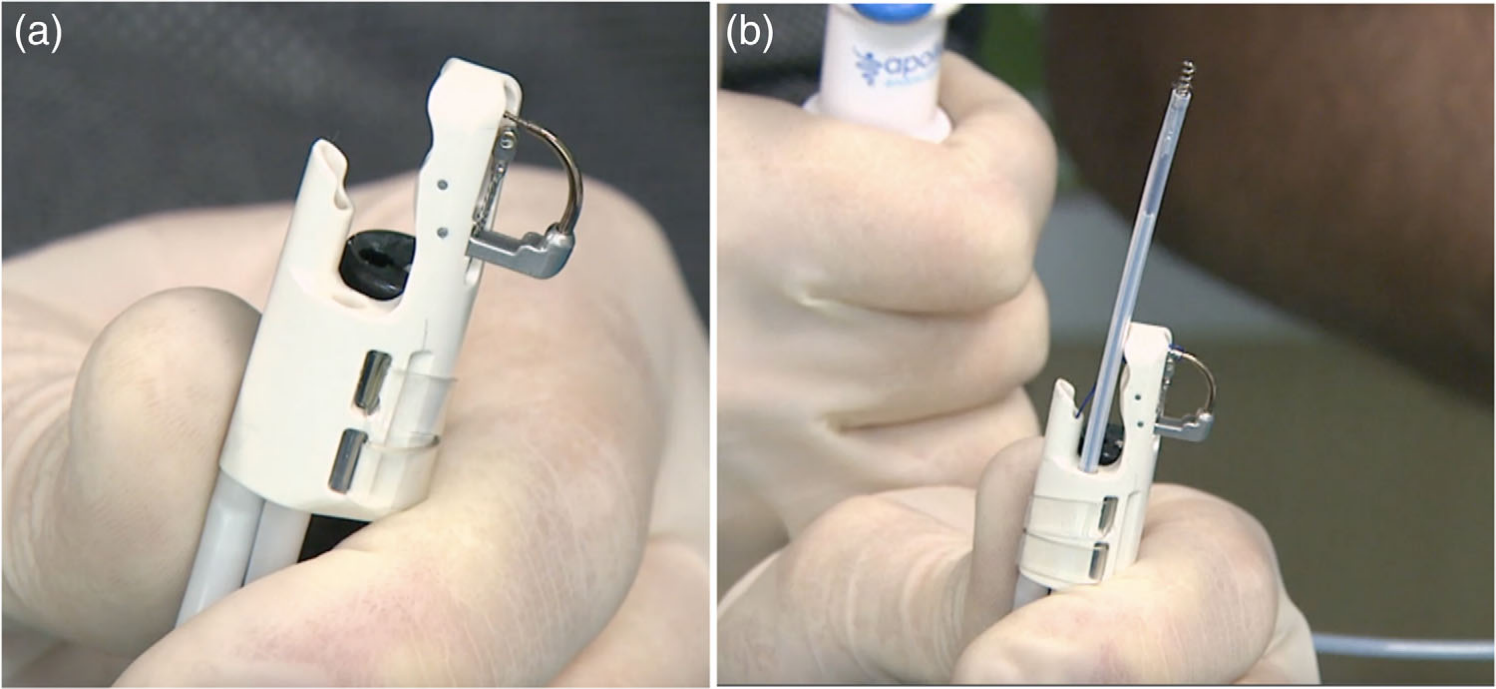
(a) The helix offset is smaller, and the exit port is located at the side. (b) The tissue helix appears at the central axis of the needle‐driver assembly.

The single‐channel suturing device differs from its predecessor in its design and assembly. The device is mounted to the side of the endoscope as compared to enface distal attachment with a second‐generation device. The tower height (19.8 vs.18.4 mm) is longer, and the diameter (16.4 vs. 15.8 mm) of the needle driver device at the distal endcap is wider than the second‐generation device (Figure 3). The helix exit port is located at the side, and when the helix is introduced through the working channel, it appears at the central axis of the needle‐driver assembly (Figure 4). The device has several advantages to highlight. The flexibility is improved markedly when using a single‐channel endoscope. The non‐metallic distal endcap is less traumatic to the mucosa, resulting in less contact bleeding. The side‐mounted design enhances visibility during suturing and does not interfere with the field of view. The extended tower height enables capturing a large amount of tissue within the full‐thickness bites. Additionally, the suctioning ability is improved, allowing quick clearing of debris and blood clots during the procedure .^12^

Despite the improved design, we encountered certain challenges when using the device. Unlike the second‐generation system, assembling the device is time‐consuming and required added skills to fix the distal endcap. Based on our experience, we found there was a learning curve, and the average time required for device set‐up reduced from 20 to 8 min after five cases. The endcap tends to rotate and change orientation while fastening the two silicone straps. It is preferable to position the distal endoscope channel at the level of the stop sign at the endcap. Then securing the scope and the endcap with the left hand while using the right hand to tighten the straps prevented malrotation. Second, when tightening the straps, it was critical to ensure that the two straps were evenly spaced and nonoverlapping and there were no gaps between the straps and the endoscope. Improper fixing of the distal endcap would result in a change in the orientation during the procedure. In our series, we noticed that the distal endcap orientation changed in two patients during endoscope insertion. In the third case, the endcap detached halfway through the procedure requiring us to remove the scope and re‐enter with a new device to complete the ESG. It was still possible to continue with ESG even if the orientation of the endcap changed. However, it required additional maneuvering of the endoscope to achieve full‐thickness bites. Third, adequate lubrication between the endoscope and the external sheet was needed to reduce friction and improve endoscope flexibility. Fourth, in a flexed endoscope position, resistance was encountered when advancing the cinch catheter and bringing the tissues together. In such instances, we straightened the scope and advanced the cinch catheter along the axis of the suture. This helped to converge the tissues together and cut the suture quickly. Lastly, the light reflection from the white distal endcap caused excessive brightness and halation. Using the newer black endcap design or changing the iris mode in the endoscope to peak could overcome this issue. In some instances, adherence of blood clots between the distal endcap and the endoscope obscured visualization. When refractory to flushing, we removed the device and washed the clots away to re‐establish good visualization. Nonetheless, we performed all the ESG procedures successfully. A weight loss of 5%–15% improves all obesity‐related comorbidities, enhances the quality of life, and decreases mortality.^21^, ^22^ In our study, 83.3%, 72.2%, and 56% achieved more than 5%, 10%, and 15% weight loss.

Our study has several strengths and certain limitations. We present the first multicenter experience on a single‐channel suturing device and have shared information on the ESG technique, challenges with the device and potential solutions, and the weight loss outcomes at 1 year. Our patients were followed up by a multidisciplinary team and therapeutic endoscopists performed all the ESG procedures. Our study is limited by its retrospective design, short follow‐up, and lack of a control group. Although multiple endoscopists performed the procedure, their differing expertise with the single‐channel suturing device could be seen as a limitation. We included all the consecutive patients who underwent ESG, and there was no selection bias. We recorded the outcome data prospectively in our database. We noticed follow‐up loss in some patients. Failure to follow up is still a significant issue in obesity management despite rigorous patient selection. The ongoing coronavirus disease 2019 pandemic added further challenges in delivering face‐to‐face follow‐up care and participation in physiotherapy sessions. Despite the restrictions, we observed most patients achieved significant weight loss.

In summary, the availability of endoscopic suturing systems is paving the way for a paradigm shift in the approach to obesity and endosurgical interventions. ESG using the single‐channel suturing system is safe and effective in inducing weight loss in patients with obesity and meets the threshold recommended by the American Society for Gastrointestinal Endoscopy and ASMBS.^6^, ^23^ The ability to mount the suturing system on most endoscopy platforms would widen the access to treatment. However, training the assistant on the device assembly and ensuring the correct orientation of the distal endcap is vital for a successful procedure. As the list of applications for endoscopic suturing is expanding, more evidence is needed on the safety and utility of the single‐channel suturing device.

## CONFLICT OF INTEREST STATEMENT

Dr. Ravishankar Asokkumar is a consultant for Apollo Endosurgery, USA. The rest of the authors have no conflict of interest.
